# 1-[2,4,6-Trimethyl-3,5-bis­(4-oxopiperidin-1-ylmeth­yl)benz­yl]piperidin-4-one

**DOI:** 10.1107/S1600536810016570

**Published:** 2010-05-12

**Authors:** K. Rajesh, V. Vijayakumar, S. Sarveswari, T. Narasimhamurthy, Edward R. T. Tiekink

**Affiliations:** a Organic Chemistry Division, School of Advanced Sciences, VIT University, Vellore 632 014, India; b Materials Research Centre, Indian Institute of Science, Bengaluru 560 012, India; c Department of Chemistry, University of Malaya, 50603 Kuala Lumpur, Malaysia

## Abstract

In the structure of the title compound, C_27_H_39_N_3_O_3_, each of the (4-oxopiperidin-1-yl)methyl residues adopts a flattened chair conformation (with the N and carbonyl groups being oriented to either side of the central C_4_ plane) and they occupy positions approximately orthogonal to the central benzene ring [C_benzene_—C—C_methyl­ene_—N torsion angles 103.4 (2), −104.4 (3) and 71.9 (3)°]; further, two of these residues are oriented to one side of the central benzene ring with the third to the other side. In the crystal packing, supra­molecular layers in the *ab* plane are sustained by C—H⋯O inter­actions.

## Related literature

For background to the biological significance of piperidin-4-one and analogous pyran and thio­pyran species, see: El-Subbagh *et al.* (2000 ▶); Ganellin *et al.* (1965 ▶); Hagenbach & Gysin (1952 ▶); Ileana *et al.* (1985 ▶); Mokio *et al.* (1989 ▶); Pathak *et al.* (2007 ▶). For a related structure, see: Vijayakumar *et al.* (2010 ▶).
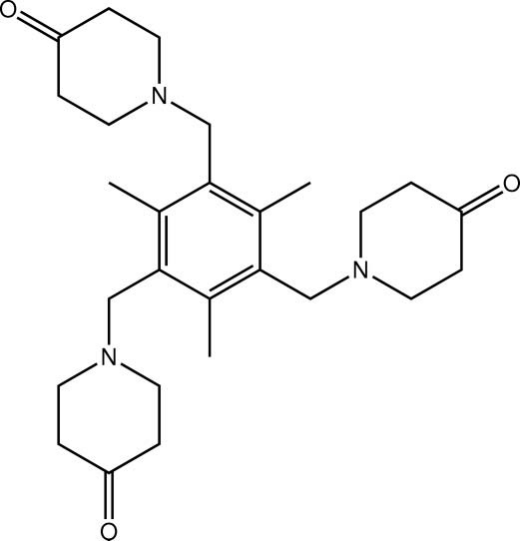


## Experimental

### 

#### Crystal data

C_27_H_39_N_3_O_3_
*M* *_r_* = 453.61
Triclinic, 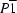

*a* = 7.9315 (16) Å
*b* = 12.449 (3) Å
*c* = 14.618 (3) Å
α = 67.641 (3)°
β = 87.749 (4)°
γ = 73.630 (3)°
*V* = 1277.0 (5) Å^3^
*Z* = 2
Mo *K*α radiation
μ = 0.08 mm^−1^
*T* = 293 K
0.28 × 0.21 × 0.17 mm

#### Data collection

Bruker SMART APEX CCD diffractometer
Absorption correction: multi-scan (*SADABS*; Sheldrick, 1998 ▶) *T*_min_ = 0.981, *T*_max_ = 0.987
12284 measured reflections
4490 independent reflections
3008 reflections with *I* > 2σ(*I*)
*R*_int_ = 0.026

#### Refinement

*R*[*F* ^2^ > 2σ(*F* ^2^)] = 0.062
*wR*(*F* ^2^) = 0.183
*S* = 1.02
4490 reflections
301 parameters
H-atom parameters constrained
Δρ_max_ = 0.26 e Å^−3^
Δρ_min_ = −0.14 e Å^−3^

### 

Data collection: *SMART* (Bruker, 2001 ▶); cell refinement: *SAINT* (Bruker, 2001 ▶); data reduction: *SAINT*; program(s) used to solve structure: *SHELXS97* (Sheldrick, 2008 ▶); program(s) used to refine structure: *SHELXL97* (Sheldrick, 2008 ▶); molecular graphics: *ORTEP-3* (Farrugia, 1997 ▶) and *DIAMOND* (Brandenburg, 2006 ▶); software used to prepare material for publication: *publCIF* (Westrip, 2010 ▶).

## Supplementary Material

Crystal structure: contains datablocks global, I. DOI: 10.1107/S1600536810016570/hg2682sup1.cif

Structure factors: contains datablocks I. DOI: 10.1107/S1600536810016570/hg2682Isup2.hkl

Additional supplementary materials:  crystallographic information; 3D view; checkCIF report

## Figures and Tables

**Table 1:** Hydrogen-bond geometry (Å, °)

*D*—H⋯*A*	*D*—H	H⋯*A*	*D*⋯*A*	*D*—H⋯*A*
C20—H20a⋯N3	0.96	2.46	3.184 (4)	132
C9—H9a⋯O2^i^	0.97	2.60	3.412 (5)	142
C21—H21b⋯O3^ii^	0.97	2.48	3.252 (4)	136

Symmetry codes: (i) 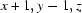; (ii) 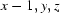.

**Fig. 1.**
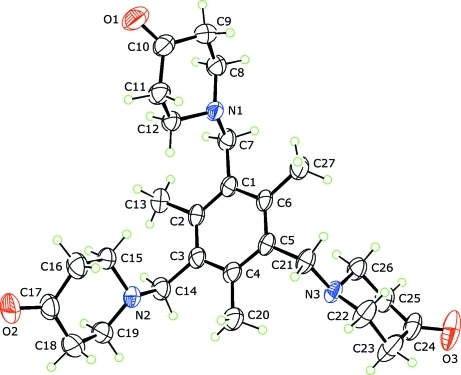
The molecular structure of (I) showing the atom-labelling scheme and displacement ellipsoids at the 35% probability level.

**Fig. 2.**
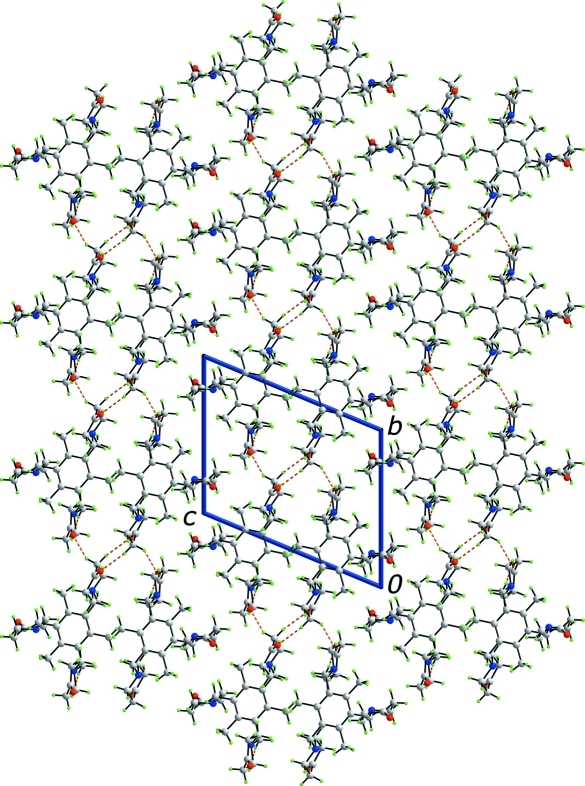
A view in projection down the a axis of the unit cell contents in (I) highlighting the stacking of layers (mediated by C–H···O contacts, shown as orange dashed lines) along the c axis.

## supplementary crystallographic information

### Comment

Piperidin-4-one and their analogous pyran and thiopyran species attract interest owing to their biological properties, viz. anti-viral, anti-tumour (El-Subbagh *et al.*, 2000), central nervous system (Ganellin *et al.*, 1965), local anesthetic (Hagenbach *et al.*, 1952), anti-cancer (Ileana *et al.*, 1985), and anti-microbial (Mokio *et al.*, 1989; Pathak *et al.*, 2007) activities. As a continuation of structural studies of piperidine-4-ones (Vijayakumar *et al.*, 2010), the title compound, (I), was synthesised and characterised by X-ray crystallography.

In compound (I), Fig. 1, the (4-oxopiperidin-1-yl)methyl residues containing the N1 and N2 atoms lie to one side of the central benzene ring and that with the N3 atom to the other. Owing to the presence of methyl substituents on either side of each 4-oxopiperidin-1-yl)methyl residue, the piperidin-4-one rings adopt side-on conformations to minimise steric interactions so that the N atoms occupy positions approximately normal to the plane through the benzene rings. This is quantified by the C2–C1–C7–N1 [103.4 (2) °], C2–C3–C14–N2 [-104.4 (3) °], and C4–C5–C21–N3 [71.9 (3) °] torsion angles. Each of the six-membered piperidin-4-one rings adopts a slightly flattened chair conformation with the N and carbonyl groups lying to either side of the central C_4_ plane in each case. Only the amine-N3 atom forms a significant intra- or inter-molecular interaction, i.e. an intramolecular C–H···N contact, Table 1. In the crystal packing, molecules are sustained into layers by C–H···O interactions; Table 1. Layers are formed in the *ab* plane and stack along the *c* axis, Fig. 2.

### Experimental

To a suspension of 1.5 equiv. of 4-piperidone hydrochloride monohydrate in benzene (20 ml), 3.0 equiv of K_2_CO_3_ was added. After stirring well for 30 min, 2,4,6-tris(bromomethyl)mesitylene (0.5 equiv) in benzene (10 ml) was added, followed by refluxing for 10 h. The completion of reaction was monitored by TLC. The reaction mixture was then allowed to cool to room temperature, filtered to remove the insoluble solids and then the filter cake was washed with dichloromethane. Excess solvents were removed under reduced pressure and the obtained crude product was purified by crystallization using 1:1 ratio of chloroform and methanol; m.pt. 483 K.

### Refinement

Carbon-bound H-atoms were placed in calculated positions (C—H 0.96 to 0.97 Å) and were included in the refinement in the riding model approximation, with *U*_iso_(H) set to 1.2–1.5*U*_equiv_(C).

### Crystal data

**Table:**

C_27_H_39_N_3_O_3_	*Z* = 2
*M**_r_* = 453.61	*F*(000) = 492
Triclinic, *P*1	*D*_x_ = 1.180 Mg m^−^^3^
Hall symbol: -P 1	Mo *K*α radiation, λ = 0.71073 Å
*a* = 7.9315 (16) Å	Cell parameters from 969 reflections
*b* = 12.449 (3) Å	θ = 2.9–21.9°
*c* = 14.618 (3) Å	µ = 0.08 mm^−^^1^
α = 67.641 (3)°	*T* = 293 K
β = 87.749 (4)°	Block, colourless
γ = 73.630 (3)°	0.28 × 0.21 × 0.17 mm
*V* = 1277.0 (5) Å^3^	

### Data collection

**Table:**

Bruker SMART APEX CCD diffractometer	4490 independent reflections
Radiation source: fine-focus sealed tube	3008 reflections with *I* > 2σ(*I*)
graphite	*R*_int_ = 0.026
ω scans	θ_max_ = 25.0°, θ_min_ = 1.5°
Absorption correction: multi-scan (*SADABS*; Sheldrick, 1998)	*h* = −9→9
*T*_min_ = 0.981, *T*_max_ = 0.987	*k* = −14→14
12284 measured reflections	*l* = −17→17

### Refinement

**Table:**

Refinement on *F*^2^	Primary atom site location: structure-invariant direct methods
Least-squares matrix: full	Secondary atom site location: difference Fourier map
*R*[*F*^2^ > 2σ(*F*^2^)] = 0.062	Hydrogen site location: inferred from neighbouring sites
*wR*(*F*^2^) = 0.183	H-atom parameters constrained
*S* = 1.02	*w* = 1/[σ^2^(*F*_o_^2^) + (0.0929*P*)^2^ + 0.3308*P*] where *P* = (*F*_o_^2^ + 2*F*_c_^2^)/3
4490 reflections	(Δ/σ)_max_ = 0.007
301 parameters	Δρ_max_ = 0.26 e Å^−^^3^
0 restraints	Δρ_min_ = −0.14 e Å^−^^3^

### Special details

**Table:**

Geometry. All s.u.'s (except the s.u. in the dihedral angle between two l.s. planes) are estimated using the full covariance matrix. The cell s.u.'s are taken into account individually in the estimation of s.u.'s in distances, angles and torsion angles; correlations between s.u.'s in cell parameters are only used when they are defined by crystal symmetry. An approximate (isotropic) treatment of cell s.u.'s is used for estimating s.u.'s involving l.s. planes.
Refinement. Refinement of *F*^2^ against ALL reflections. The weighted *R*-factor wR and goodness of fit *S* are based on *F*^2^, conventional *R*-factors *R* are based on *F*, with *F* set to zero for negative *F*^2^. The threshold expression of *F*^2^ > 2σ(*F*^2^) is used only for calculating *R*-factors(gt) etc. and is not relevant to the choice of reflections for refinement. *R*-factors based on *F*^2^ are statistically about twice as large as those based on *F*, and *R*- factors based on ALL data will be even larger.

### Fractional atomic coordinates and isotropic or equivalent isotropic displacement parameters (Å^2^)

**Table:**

	*x*	*y*	*z*	*U*_iso_*/*U*_eq_	
O1	−0.1090 (3)	−0.3541 (2)	0.4155 (2)	0.1134 (9)	
O2	−0.5097 (3)	0.4895 (2)	0.2615 (2)	0.1089 (8)	
O3	1.1430 (3)	0.1907 (3)	−0.0499 (2)	0.1334 (11)	
N1	0.2978 (2)	−0.20717 (17)	0.36948 (14)	0.0539 (5)	
N2	0.0198 (2)	0.35113 (19)	0.26602 (16)	0.0603 (6)	
N3	0.6691 (2)	0.18892 (18)	0.05220 (14)	0.0558 (5)	
C1	0.4104 (3)	−0.0297 (2)	0.33087 (17)	0.0502 (6)	
C2	0.3290 (3)	0.0681 (2)	0.35901 (17)	0.0511 (6)	
C3	0.3003 (3)	0.1875 (2)	0.29004 (19)	0.0532 (6)	
C4	0.3618 (3)	0.2095 (2)	0.19514 (18)	0.0545 (6)	
C5	0.4516 (3)	0.1122 (2)	0.16894 (17)	0.0520 (6)	
C6	0.4721 (3)	−0.0076 (2)	0.23580 (18)	0.0511 (6)	
C7	0.4264 (3)	−0.1596 (2)	0.39930 (19)	0.0592 (7)	
H7A	0.5444	−0.2101	0.3991	0.071*	
H7B	0.4088	−0.1636	0.4664	0.071*	
C8	0.3258 (3)	−0.3350 (2)	0.4315 (2)	0.0705 (8)	
H8A	0.3095	−0.3439	0.4999	0.085*	
H8B	0.4462	−0.3799	0.4284	0.085*	
C9	0.1999 (4)	−0.3884 (3)	0.3990 (3)	0.0792 (9)	
H9A	0.2340	−0.3963	0.3369	0.095*	
H9B	0.2100	−0.4689	0.4483	0.095*	
C10	0.0127 (4)	−0.3128 (3)	0.3857 (2)	0.0704 (8)	
C11	−0.0149 (3)	−0.1805 (2)	0.3333 (2)	0.0684 (7)	
H11A	−0.1333	−0.1373	0.3415	0.082*	
H11B	−0.0036	−0.1616	0.2630	0.082*	
C12	0.1175 (3)	−0.1385 (2)	0.37301 (19)	0.0580 (6)	
H12A	0.1019	−0.0531	0.3340	0.070*	
H12B	0.0960	−0.1479	0.4410	0.070*	
C13	0.2677 (3)	0.0440 (3)	0.46323 (19)	0.0677 (7)	
H13A	0.1498	0.0375	0.4643	0.102*	
H13B	0.2698	0.1097	0.4819	0.102*	
H13C	0.3448	−0.0303	0.5091	0.102*	
C14	0.2003 (3)	0.2953 (2)	0.3141 (2)	0.0655 (7)	
H14A	0.1946	0.2693	0.3854	0.079*	
H14B	0.2641	0.3556	0.2929	0.079*	
C15	−0.0916 (3)	0.2725 (2)	0.3062 (2)	0.0587 (6)	
H15A	−0.1012	0.2573	0.3761	0.070*	
H15B	−0.0373	0.1953	0.3004	0.070*	
C16	−0.2748 (3)	0.3271 (3)	0.2531 (2)	0.0770 (8)	
H16A	−0.2675	0.3275	0.1866	0.092*	
H16B	−0.3491	0.2767	0.2881	0.092*	
C17	−0.3573 (4)	0.4534 (3)	0.2471 (2)	0.0750 (8)	
C18	−0.2391 (4)	0.5317 (3)	0.2223 (3)	0.1045 (12)	
H18A	−0.2927	0.6024	0.2380	0.125*	
H18B	−0.2249	0.5597	0.1516	0.125*	
C19	−0.0583 (4)	0.4656 (3)	0.2789 (3)	0.0860 (10)	
H19A	0.0189	0.5170	0.2551	0.103*	
H19B	−0.0698	0.4493	0.3488	0.103*	
C20	0.3263 (3)	0.3379 (3)	0.1196 (2)	0.0757 (8)	
H20A	0.4242	0.3442	0.0792	0.114*	
H20B	0.3107	0.3926	0.1532	0.114*	
H20C	0.2214	0.3586	0.0784	0.114*	
C21	0.5171 (3)	0.1390 (3)	0.06593 (18)	0.0628 (7)	
H21A	0.5507	0.0648	0.0536	0.075*	
H21B	0.4215	0.1966	0.0174	0.075*	
C22	0.6971 (4)	0.2351 (3)	−0.0532 (2)	0.0842 (10)	
H22A	0.5887	0.2925	−0.0905	0.101*	
H22B	0.7295	0.1686	−0.0758	0.101*	
C23	0.8419 (4)	0.2969 (4)	−0.0716 (3)	0.1011 (12)	
H23A	0.8620	0.3253	−0.1416	0.121*	
H23B	0.8064	0.3665	−0.0530	0.121*	
C24	1.0056 (4)	0.2109 (3)	−0.0130 (2)	0.0797 (9)	
C25	0.9854 (3)	0.1473 (3)	0.0935 (2)	0.0755 (8)	
H25A	0.9698	0.2031	0.1269	0.091*	
H25B	1.0913	0.0811	0.1235	0.091*	
C26	0.8270 (3)	0.0975 (2)	0.1068 (2)	0.0644 (7)	
H26A	0.8527	0.0309	0.0845	0.077*	
H26B	0.8071	0.0659	0.1767	0.077*	
C27	0.5526 (3)	−0.1134 (3)	0.2056 (2)	0.0691 (8)	
H27A	0.4736	−0.1128	0.1572	0.104*	
H27B	0.5726	−0.1879	0.2628	0.104*	
H27C	0.6625	−0.1067	0.1777	0.104*	

### Atomic displacement parameters (Å^2^)

**Table:**

	*U*^11^	*U*^22^	*U*^33^	*U*^12^	*U*^13^	*U*^23^
O1	0.0747 (14)	0.0842 (16)	0.159 (2)	−0.0394 (13)	0.0274 (15)	−0.0133 (15)
O2	0.0508 (12)	0.1078 (18)	0.160 (2)	−0.0044 (12)	0.0228 (13)	−0.0575 (16)
O3	0.0656 (14)	0.245 (3)	0.131 (2)	−0.0694 (18)	0.0524 (14)	−0.105 (2)
N1	0.0428 (10)	0.0519 (12)	0.0582 (12)	−0.0119 (9)	0.0099 (9)	−0.0137 (10)
N2	0.0401 (10)	0.0613 (13)	0.0751 (14)	−0.0127 (9)	0.0124 (9)	−0.0238 (11)
N3	0.0351 (10)	0.0699 (13)	0.0532 (12)	−0.0180 (9)	0.0068 (8)	−0.0122 (10)
C1	0.0308 (11)	0.0641 (15)	0.0553 (14)	−0.0161 (10)	0.0028 (10)	−0.0210 (12)
C2	0.0303 (11)	0.0717 (17)	0.0548 (14)	−0.0155 (11)	0.0042 (10)	−0.0276 (13)
C3	0.0307 (11)	0.0663 (16)	0.0676 (16)	−0.0172 (11)	0.0049 (10)	−0.0288 (13)
C4	0.0301 (11)	0.0641 (16)	0.0664 (16)	−0.0170 (11)	0.0025 (10)	−0.0193 (13)
C5	0.0289 (11)	0.0755 (17)	0.0561 (14)	−0.0225 (11)	0.0062 (10)	−0.0251 (13)
C6	0.0305 (11)	0.0688 (16)	0.0632 (15)	−0.0209 (11)	0.0105 (10)	−0.0312 (13)
C7	0.0401 (12)	0.0672 (17)	0.0615 (15)	−0.0099 (11)	0.0024 (11)	−0.0191 (13)
C8	0.0538 (15)	0.0587 (17)	0.0804 (19)	−0.0076 (13)	0.0117 (13)	−0.0137 (14)
C9	0.078 (2)	0.0552 (17)	0.100 (2)	−0.0240 (15)	0.0208 (17)	−0.0225 (16)
C10	0.0621 (17)	0.0688 (18)	0.0790 (19)	−0.0278 (14)	0.0131 (14)	−0.0213 (15)
C11	0.0518 (15)	0.0663 (17)	0.0783 (18)	−0.0207 (13)	0.0024 (13)	−0.0157 (14)
C12	0.0443 (13)	0.0559 (15)	0.0659 (16)	−0.0132 (11)	0.0056 (11)	−0.0161 (12)
C13	0.0547 (15)	0.0854 (19)	0.0636 (17)	−0.0163 (14)	0.0091 (12)	−0.0327 (15)
C14	0.0443 (14)	0.0750 (18)	0.0871 (19)	−0.0201 (13)	0.0092 (13)	−0.0403 (15)
C15	0.0414 (13)	0.0625 (16)	0.0712 (16)	−0.0131 (11)	0.0062 (11)	−0.0262 (13)
C16	0.0466 (15)	0.087 (2)	0.098 (2)	−0.0117 (14)	0.0010 (14)	−0.0419 (18)
C17	0.0468 (15)	0.080 (2)	0.085 (2)	−0.0035 (14)	0.0067 (13)	−0.0281 (16)
C18	0.0654 (19)	0.064 (2)	0.153 (3)	−0.0025 (16)	0.018 (2)	−0.022 (2)
C19	0.0603 (17)	0.0647 (19)	0.136 (3)	−0.0224 (15)	0.0241 (18)	−0.0402 (19)
C20	0.0501 (15)	0.0744 (19)	0.086 (2)	−0.0166 (13)	0.0093 (14)	−0.0149 (16)
C21	0.0425 (13)	0.0917 (19)	0.0569 (15)	−0.0282 (13)	0.0075 (11)	−0.0257 (14)
C22	0.0580 (16)	0.116 (3)	0.0561 (17)	−0.0340 (17)	0.0056 (13)	−0.0031 (16)
C23	0.079 (2)	0.122 (3)	0.086 (2)	−0.052 (2)	0.0246 (18)	−0.008 (2)
C24	0.0575 (17)	0.123 (3)	0.092 (2)	−0.0536 (18)	0.0352 (16)	−0.060 (2)
C25	0.0408 (14)	0.105 (2)	0.084 (2)	−0.0235 (14)	0.0092 (13)	−0.0388 (18)
C26	0.0408 (13)	0.0757 (18)	0.0666 (16)	−0.0169 (12)	0.0054 (11)	−0.0166 (14)
C27	0.0569 (15)	0.0853 (19)	0.0825 (19)	−0.0324 (14)	0.0234 (14)	−0.0440 (16)

### Geometric parameters (Å, °)

**Table:**

O1—C10	1.209 (3)	C13—H13A	0.9600
O2—C17	1.205 (3)	C13—H13B	0.9600
O3—C24	1.202 (3)	C13—H13C	0.9600
N1—C12	1.455 (3)	C14—H14A	0.9700
N1—C8	1.457 (3)	C14—H14B	0.9700
N1—C7	1.468 (3)	C15—C16	1.518 (3)
N2—C15	1.447 (3)	C15—H15A	0.9700
N2—C19	1.465 (3)	C15—H15B	0.9700
N2—C14	1.474 (3)	C16—C17	1.493 (4)
N3—C26	1.444 (3)	C16—H16A	0.9700
N3—C22	1.457 (3)	C16—H16B	0.9700
N3—C21	1.480 (3)	C17—C18	1.477 (4)
C1—C6	1.407 (3)	C18—C19	1.526 (4)
C1—C2	1.409 (3)	C18—H18A	0.9700
C1—C7	1.515 (3)	C18—H18B	0.9700
C2—C3	1.402 (3)	C19—H19A	0.9700
C2—C13	1.524 (3)	C19—H19B	0.9700
C3—C4	1.404 (3)	C20—H20A	0.9600
C3—C14	1.513 (3)	C20—H20B	0.9600
C4—C5	1.402 (3)	C20—H20C	0.9600
C4—C20	1.511 (4)	C21—H21A	0.9700
C5—C6	1.405 (3)	C21—H21B	0.9700
C5—C21	1.517 (3)	C22—C23	1.516 (4)
C6—C27	1.511 (3)	C22—H22A	0.9700
C7—H7A	0.9700	C22—H22B	0.9700
C7—H7B	0.9700	C23—C24	1.474 (5)
C8—C9	1.522 (4)	C23—H23A	0.9700
C8—H8A	0.9700	C23—H23B	0.9700
C8—H8B	0.9700	C24—C25	1.480 (4)
C9—C10	1.491 (4)	C25—C26	1.525 (3)
C9—H9A	0.9700	C25—H25A	0.9700
C9—H9B	0.9700	C25—H25B	0.9700
C10—C11	1.482 (4)	C26—H26A	0.9700
C11—C12	1.517 (3)	C26—H26B	0.9700
C11—H11A	0.9700	C27—H27A	0.9600
C11—H11B	0.9700	C27—H27B	0.9600
C12—H12A	0.9700	C27—H27C	0.9600
C12—H12B	0.9700		
			
C12—N1—C8	109.97 (18)	N2—C15—C16	112.2 (2)
C12—N1—C7	111.77 (19)	N2—C15—H15A	109.2
C8—N1—C7	110.82 (19)	C16—C15—H15A	109.2
C15—N2—C19	109.09 (19)	N2—C15—H15B	109.2
C15—N2—C14	112.1 (2)	C16—C15—H15B	109.2
C19—N2—C14	109.6 (2)	H15A—C15—H15B	107.9
C26—N3—C22	109.5 (2)	C17—C16—C15	112.2 (2)
C26—N3—C21	111.39 (19)	C17—C16—H16A	109.2
C22—N3—C21	108.5 (2)	C15—C16—H16A	109.2
C6—C1—C2	120.0 (2)	C17—C16—H16B	109.2
C6—C1—C7	118.4 (2)	C15—C16—H16B	109.2
C2—C1—C7	121.5 (2)	H16A—C16—H16B	107.9
C3—C2—C1	119.8 (2)	O2—C17—C18	122.0 (3)
C3—C2—C13	120.1 (2)	O2—C17—C16	122.6 (3)
C1—C2—C13	120.0 (2)	C18—C17—C16	115.4 (2)
C2—C3—C4	120.0 (2)	C17—C18—C19	112.1 (3)
C2—C3—C14	121.7 (2)	C17—C18—H18A	109.2
C4—C3—C14	118.3 (2)	C19—C18—H18A	109.2
C5—C4—C3	120.2 (2)	C17—C18—H18B	109.2
C5—C4—C20	119.6 (2)	C19—C18—H18B	109.2
C3—C4—C20	120.2 (2)	H18A—C18—H18B	107.9
C4—C5—C6	120.0 (2)	N2—C19—C18	111.2 (3)
C4—C5—C21	118.9 (2)	N2—C19—H19A	109.4
C6—C5—C21	121.0 (2)	C18—C19—H19A	109.4
C5—C6—C1	119.8 (2)	N2—C19—H19B	109.4
C5—C6—C27	120.9 (2)	C18—C19—H19B	109.4
C1—C6—C27	119.2 (2)	H19A—C19—H19B	108.0
N1—C7—C1	112.09 (18)	C4—C20—H20A	109.5
N1—C7—H7A	109.2	C4—C20—H20B	109.5
C1—C7—H7A	109.2	H20A—C20—H20B	109.5
N1—C7—H7B	109.2	C4—C20—H20C	109.5
C1—C7—H7B	109.2	H20A—C20—H20C	109.5
H7A—C7—H7B	107.9	H20B—C20—H20C	109.5
N1—C8—C9	112.0 (2)	N3—C21—C5	113.1 (2)
N1—C8—H8A	109.2	N3—C21—H21A	108.9
C9—C8—H8A	109.2	C5—C21—H21A	108.9
N1—C8—H8B	109.2	N3—C21—H21B	108.9
C9—C8—H8B	109.2	C5—C21—H21B	108.9
H8A—C8—H8B	107.9	H21A—C21—H21B	107.8
C10—C9—C8	112.8 (2)	N3—C22—C23	110.2 (3)
C10—C9—H9A	109.0	N3—C22—H22A	109.6
C8—C9—H9A	109.0	C23—C22—H22A	109.6
C10—C9—H9B	109.0	N3—C22—H22B	109.6
C8—C9—H9B	109.0	C23—C22—H22B	109.6
H9A—C9—H9B	107.8	H22A—C22—H22B	108.1
O1—C10—C11	121.5 (3)	C24—C23—C22	109.8 (3)
O1—C10—C9	123.7 (3)	C24—C23—H23A	109.7
C11—C10—C9	114.8 (2)	C22—C23—H23A	109.7
C10—C11—C12	111.3 (2)	C24—C23—H23B	109.7
C10—C11—H11A	109.4	C22—C23—H23B	109.7
C12—C11—H11A	109.4	H23A—C23—H23B	108.2
C10—C11—H11B	109.4	O3—C24—C23	122.3 (3)
C12—C11—H11B	109.4	O3—C24—C25	122.9 (3)
H11A—C11—H11B	108.0	C23—C24—C25	114.8 (2)
N1—C12—C11	111.6 (2)	C24—C25—C26	110.8 (2)
N1—C12—H12A	109.3	C24—C25—H25A	109.5
C11—C12—H12A	109.3	C26—C25—H25A	109.5
N1—C12—H12B	109.3	C24—C25—H25B	109.5
C11—C12—H12B	109.3	C26—C25—H25B	109.5
H12A—C12—H12B	108.0	H25A—C25—H25B	108.1
C2—C13—H13A	109.5	N3—C26—C25	112.1 (2)
C2—C13—H13B	109.5	N3—C26—H26A	109.2
H13A—C13—H13B	109.5	C25—C26—H26A	109.2
C2—C13—H13C	109.5	N3—C26—H26B	109.2
H13A—C13—H13C	109.5	C25—C26—H26B	109.2
H13B—C13—H13C	109.5	H26A—C26—H26B	107.9
N2—C14—C3	112.7 (2)	C6—C27—H27A	109.5
N2—C14—H14A	109.1	C6—C27—H27B	109.5
C3—C14—H14A	109.1	H27A—C27—H27B	109.5
N2—C14—H14B	109.1	C6—C27—H27C	109.5
C3—C14—H14B	109.1	H27A—C27—H27C	109.5
H14A—C14—H14B	107.8	H27B—C27—H27C	109.5
			
C6—C1—C2—C3	3.8 (3)	O1—C10—C11—C12	133.7 (3)
C7—C1—C2—C3	−173.44 (19)	C9—C10—C11—C12	−45.6 (4)
C6—C1—C2—C13	−177.93 (19)	C8—N1—C12—C11	−61.6 (3)
C7—C1—C2—C13	4.8 (3)	C7—N1—C12—C11	174.8 (2)
C1—C2—C3—C4	−3.7 (3)	C10—C11—C12—N1	54.7 (3)
C13—C2—C3—C4	178.0 (2)	C15—N2—C14—C3	68.0 (3)
C1—C2—C3—C14	174.9 (2)	C19—N2—C14—C3	−170.7 (2)
C13—C2—C3—C14	−3.3 (3)	C2—C3—C14—N2	−104.4 (3)
C2—C3—C4—C5	0.1 (3)	C4—C3—C14—N2	74.3 (3)
C14—C3—C4—C5	−178.57 (19)	C19—N2—C15—C16	61.4 (3)
C2—C3—C4—C20	178.1 (2)	C14—N2—C15—C16	−177.0 (2)
C14—C3—C4—C20	−0.6 (3)	N2—C15—C16—C17	−51.2 (3)
C3—C4—C5—C6	3.4 (3)	C15—C16—C17—O2	−137.8 (3)
C20—C4—C5—C6	−174.6 (2)	C15—C16—C17—C18	42.1 (4)
C3—C4—C5—C21	−179.76 (19)	O2—C17—C18—C19	136.7 (3)
C20—C4—C5—C21	2.2 (3)	C16—C17—C18—C19	−43.1 (4)
C4—C5—C6—C1	−3.3 (3)	C15—N2—C19—C18	−61.9 (3)
C21—C5—C6—C1	179.92 (19)	C14—N2—C19—C18	175.0 (2)
C4—C5—C6—C27	174.0 (2)	C17—C18—C19—N2	52.8 (4)
C21—C5—C6—C27	−2.8 (3)	C26—N3—C21—C5	71.4 (3)
C2—C1—C6—C5	−0.3 (3)	C22—N3—C21—C5	−168.0 (2)
C7—C1—C6—C5	177.04 (19)	C4—C5—C21—N3	71.9 (3)
C2—C1—C6—C27	−177.6 (2)	C6—C5—C21—N3	−111.3 (2)
C7—C1—C6—C27	−0.3 (3)	C26—N3—C22—C23	−63.1 (3)
C12—N1—C7—C1	−61.7 (3)	C21—N3—C22—C23	175.1 (3)
C8—N1—C7—C1	175.3 (2)	N3—C22—C23—C24	57.7 (4)
C6—C1—C7—N1	−73.9 (2)	C22—C23—C24—O3	126.4 (3)
C2—C1—C7—N1	103.4 (2)	C22—C23—C24—C25	−50.7 (4)
C12—N1—C8—C9	58.7 (3)	O3—C24—C25—C26	−129.9 (3)
C7—N1—C8—C9	−177.2 (2)	C23—C24—C25—C26	47.2 (4)
N1—C8—C9—C10	−49.5 (3)	C22—N3—C26—C25	60.0 (3)
C8—C9—C10—O1	−135.9 (3)	C21—N3—C26—C25	180.0 (2)
C8—C9—C10—C11	43.5 (4)	C24—C25—C26—N3	−51.3 (3)

### Hydrogen-bond geometry (Å, °)

**Table:**

*D*—H···*A*	*D*—H	H···*A*	*D*···*A*	*D*—H···*A*
C20—H20a···N3	0.96	2.46	3.184 (4)	132
C9—H9a···O2^i^	0.97	2.60	3.412 (5)	142
C21—H21b···O3^ii^	0.97	2.48	3.252 (4)	136

Symmetry codes:  (i) *x*+1, *y*−1, *z*; (ii) *x*−1, *y*, *z*.

## References

[bb1] Brandenburg, K. (2006). *DIAMOND* Crystal Impact GbR, Bonn, Germany.

[bb2] Bruker (2001). *SMART* and *SAINT* Bruker AXS Inc., Madison, Wisconsin, USA.

[bb3] El-Subbagh, H. I., Abu-Zaid, S. M., Mahran, M. A., Badria, F. A. & Alofaid, A. M. (2000). *J. Med. Chem* **43**, 2915–2921. 10.1021/jm000038m 10956199

[bb4] Farrugia, L. J. (1997). *J. Appl. Cryst.* **30**, 565.

[bb5] Ganellin, C. R. & Spickett, R. G. (1965). *J. Med. Chem* **8**, 619–625. 10.1021/jm00329a015 5867943

[bb6] Hagenbach, R. E. & Gysin, H. (1952). *Experimentia*, **8**, 184–187. 10.1007/BF02173735 14945453

[bb7] Ileana, B., Dobre, V. & Nicluescu-Duvaz, I. (1985). *J. Prakt. Chem* **327**, 667–674.

[bb8] Mokio, I. G., Soldatenkov, A. T., Federov, V. O., Ageev, E. A., Sergeeva, N. D., Lin, S., Stashenku, E. E., Prostakov, N. S. & Andreeva, E. L. (1989). *Khim. Farm. Zh* **23**, 421–427.

[bb9] Pathak, C., Karthikeyan, S., More, K. & Vijayakumar, V. (2007). *Indian J. Heterocycl. Chem* **16**, 295–296.

[bb10] Sheldrick, G. M. (1998). *SADABS* University of Göttingen, Germany.

[bb11] Sheldrick, G. M. (2008). *Acta Cryst.* A**64**, 112–122. 10.1107/S0108767307043930 18156677

[bb12] Vijayakumar, V., Rajesh, K., Suresh, J., Narasimhamurthy, T. & Lakshman, P. L. N. (2010). *Acta Cryst.* E**66**, o170. 10.1107/S1600536809052908 PMC2980215 21580057

[bb13] Westrip, S. P. (2010). *J. Appl. Cryst.* **43** Submitted.

